# NLOS Identification in WLANs Using Deep LSTM with CNN Features

**DOI:** 10.3390/s18114057

**Published:** 2018-11-20

**Authors:** Viet-Hung Nguyen, Minh-Tuan Nguyen, Jeongsik Choi, Yong-Hwa Kim

**Affiliations:** 1 Department of Electronic Engineering, Myongji University, Yongin 449-728, Korea; nguyenviethunghc2@gmail.com(V.-H.N.); tuannguyen091095@gmail.com(M.-T.N.); 2 Intel Labs, Intel Corporation, Santa Clara, CA 95054, USA; jeongsik.choi@intel.com

**Keywords:** line-of-sight identification, channel state information, deep learning, convolutional neural network, long-short term memory model

## Abstract

Identifying channel states as line-of-sight or non-line-of-sight helps to optimize location-based services in wireless communications. The received signal strength identification and channel state information are used to estimate channel conditions for orthogonal frequency division multiplexing systems in indoor wireless local area networks. This paper proposes a joint convolutional neural network and recurrent neural network architecture to classify channel conditions. Convolutional neural networks extract the feature from frequency-domain characteristics of channel state information data and recurrent neural networks extract the feature from time-varying characteristics of received signal strength identification and channel state information between packet transmissions. The performance of the proposed methods is verified under indoor propagation environments. Experimental results show that the proposed method has a 2% improvement in classification performance over the conventional recurrent neural network model.

## 1. Introduction

Recently, location-based services such as real-time tracking, security alerts, informational services, and entertainment applications are becoming important in wireless communication infrastructures. Global positioning systems (GPSs) are the most commonly used outdoor location sensing technology [1]. However, GPSs are not suitable for indoor positioning systems due to line-of-sight (LOS) requests between the satellites and the receivers [2]. Ultra-wideband (UWB) and wireless local area network (WLAN) technologies are two major candidates for the implementation of accurate indoor positioning systems.

UWB systems use an exceedingly wide band of the radio frequency (RF) spectrum to achieve higher temporal resolution and robustness to multipath fading [3,4]. Although UWBs are expected to provide higher accuracy in indoor positioning systems than WLANs, they have the disadvantage that new communication infrastructure must be established for UWB systems [4]. WLANs are cost-efficient as they can use existing communication infrastructure. Hence, they are widely used in communication infrastructures [3].

Received signal strength indicator (RSSI)-based techniques for WLANs have been proposed for localization services [5,6]. RSSI-based systems have the advantage of using existing WLAN infrastructure. To improve localization accuracy, channel state information (CSI) is used to estimate location in WLANs. However, the reliability of communication and localization accuracy in WLANs can be seriously affected by non-line-of-sight (NLOS) propagation [7,8,9,10].

To improve WLAN localization performance, several studies have, over time, investigated how to distinguish between LOS and NLOS using handcrafted features by a series of CSI [11,12,13,14,15]. Skewness and kurtosis from CSI are used to classify LOS and NLOS environments [13,14,15]. A recurrent neural network (RNN) model with long-short term memory (LSTM) one of a deep models using RSSI and CSI has been proposed to improve the classification performance of LOS and NLOS classification [16]. However, the RNN model with LSTMs in [16] only focuses on the temporal structure of CSI data and does not contain the frequency characteristics of CSIs.

In this paper, we propose a new LOS and NLOS classification method for WLANs based on RSSI and CSI. The proposed convolutional neural network LSTM (CNNLSTM) model combines the advantages of a CNN by reducing the variance of CSI data and the ability of LSTM in modeling long-range dependencies of sequential data in a unified framework. Compared to the LSTM model [16], the proposed CNNLSTM exploits the non-temporal structure from the input by using the CNN before LSTM to learn the frequency characteristics of CSIs. The main contributions are summarized as follows. First, we introduce a state-of-the-art CNNLSTM model that provides a 2% relative improvement in classification performance over the LSTM method. Second, the proposed model achieves the highest accuracy of 96.53% compared to the CNN and LSTM models. Finally, we propose a new model that exploits absolute values of CSI data instead of using complex values of CSI so as to reduce complexity.

The rest of this paper is structured as follows. In Section 2 we briefly discuss the related works of some general deep learning models. Section 3 introduces characteristics of CSI and RSSI data from experiments. In Section 4 we describe the proposed CNNLSTM architecture to classify channel conditions. Section 5 presents the performance evaluation and network visualization. Finally, Section 6 concludes the paper and discusses future work.

## 2. Related Works

Deep learning was constituted by neural network models with deep hidden layer architectures [17]. A deep learning model produces a chain of layers that can build up increasing ranks of abstract information from the input variables to the output variables. Compared to other machine learning algorithms [18,19,20], deep learning models try to capture potential features using hidden layers automatically. In recent years, many different types of deep learning models have been introduced. This paper describes three main models: CNN, LSTM, and CNNLSTM.

A CNN was developed by preserving the spatial structure of the input data for object recognition tasks such as handwritten digit recognition [21], computer vision [22], and natural language processing [23] through the use of convolutional layers. These layers can automatically identify and generalize essential local features at varied positions in the input maps using learnable kernels; hence, it can noticeably reduce the number of parameters compared to a fully connected layer by using local connectivity and weight sharing. A CNN model extracts features with several filters in wireless communication fields such as automatic modulation classification for wireless localization [24].

An RNN is specially developed to solve problems related to sequential data such as language modeling [23] and speech recognition [25]. Compared to the fully connected neural network and CNN, RNN models have recurrence connection between time steps to consider sequential information [23,24,25,26]. An LSTM model, which is one of the most widely used RNN models, avoids the long-term dependency problem in typical RNN structures caused by the vanishing gradient with four gates to adjust information flow [27]. LSTMs were used to extract temporal features from packet transmissions of CSIs and RSSIs [16]. However, input with spatial structure cannot be well modeled using only the standard LSTM [28].

A CNNLSTM model is designed with both spatial and temporal features in mind by combining convolutional layers for latent feature extraction on input data and LSTM layers to support sequence prediction [28,29,30]. In our input data, CSIs have not only time-varying characteristics between packet transmissions but also frequency characteristics of CSI at each transmission [24]. Therefore, we consider the CNNLSTM model to use both time-varying and frequency characteristics of CSIs.

## 3. Preliminaries

In this section, we consider a system model and experimental data for commodity WLANs, where a receiver obtains the RSSI at each transmission and estimates the frequency-domain CSI of the subcarriers.

### 3.1. System Model

Let $h={\left[h\left(0\right),h\left(1\right),\ldots ,h\left(L-1\right)\right]}^{T}$ be the time-domain channel impulse response (CIR), where *L* is the number of multipath taps. The frequency- domain CIR for the kth subcarrier can be modeled as [16]

(1) $$H\left(k\right)=\sum_{l=0}^{L-1}h\left(l\right)e^{-j\frac{2\pi kl}{N}},$$

where *N* is the fast Fourier transform (FFT) size, k∈K and *K* is the fast Fourier transform (FFT) size, N−K−1 subcarriers at the edges of the spectrum are not used and the used subcarriers can be indexed by K=−K/2,…,−1,1,…,K/2, where *K* is the number of used subcarriers. At the receiver, the channel state information (CSI) for the kth subcarrier is estimated as

(2) $$\hat{H}\left(k\right)=H\left(k\right)+n\left(k\right)$$

where n(k) is complex Gaussian noise for the kth subcarrier with zero-mean and variance of $N_{0}$ [16].

In IEEE 802.11 WLANs, RSSI is provided for upper layer information. At each transmission, the RSSI is used as an indication of the received power level. RSSIs for the LOS condition are concentrated at a high value, while RSSIs for the NLOS condition are distributed over a wide range [16].

### 3.2. Experimental Data

**Figure 1:**
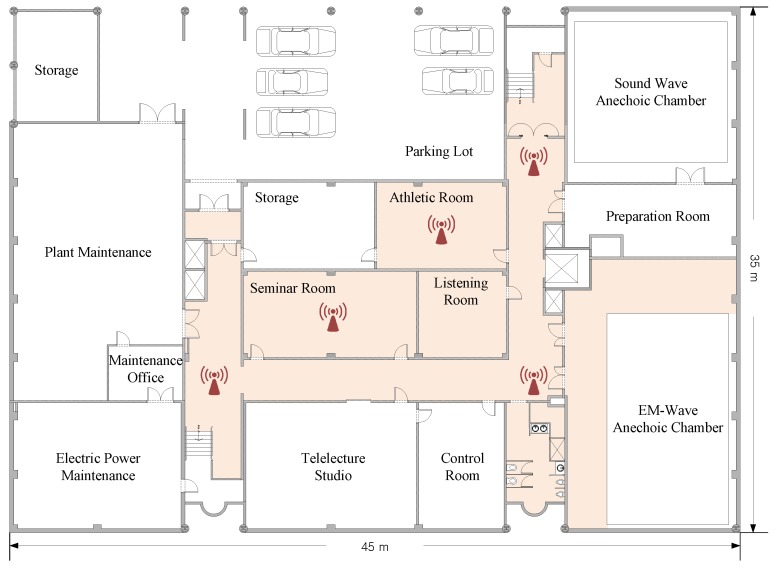
The experiment setup.

For performance comparison with the previous result, we exploited data collected at Seoul National University [16]. Figure 1 shows the layout of the measurement site, which can be considered a typical indoor office environment. For measurement campaigns, two laptops equipped with Qualcomm Atheros network interface cards (NICs) were used to capture both RSSI and CSI. The height of the transmitter and the receiver were fixed at 1.2 m. A person holding the receiver walked around the highlighted area shown in Figure 1 to collect data while recording the labels of the collected data: LOS if there was no obstacle between the transmitter and the receiver or NLOS if the direct path was blocked by the person holding the receiver or other obstacles, e.g., walls and doors.

The measurement took 4300 s to complete. During the measurement campaigns, the transmitter sent sounding packets every 10 ms and the receiver measured RSSI and CSI per packet transmission. For signal transmission, IEEE 802.11n protocol with a 20-MHz bandwidth was used, and therefore, total K=56 CSIs (i.e., full CSI report) and an RSSI were measured for each point-to-point link. Moreover, the transmitter and the receiver were equipped with two and three antennas, respectively, and six sets of CSI and three sets of RSSIs were measured during each packet transmission. Using these protocols, a total of 101,197 packet transmissions were measured under the LOS condition and 331,365 packet transmissions were measured under the NLOS condition.

## 4. Proposed CNNLSTM Model

**Figure 2:**
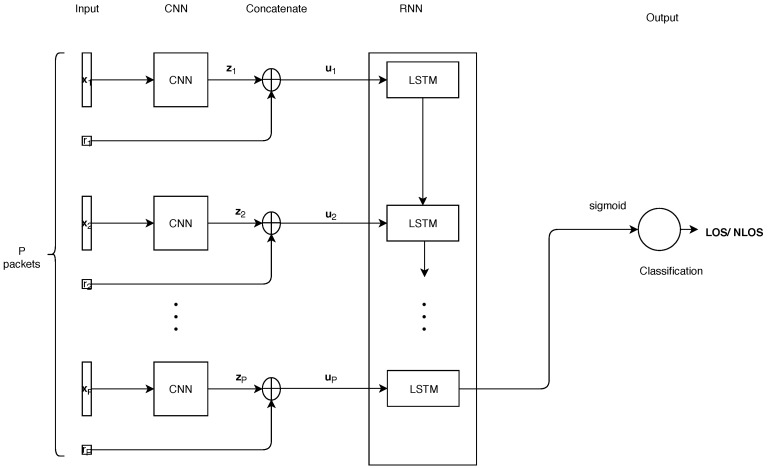
The overall framework of the proposed CNNLSTM.

To classify LOS and NLOS in WLANs, we propose a novel CNNLSTM model. Figure 2 shows the overall framework of the proposed model, that comprise CNN and RNN segments. As shown in Figure 2, the CSIs form the input signals for the CNN, while the output of CNN concatenates with the RSSIs, and feed into the LSTMs for the LOS and NLOS classification.

### 4.1. CNN Part

**Figure 3:**
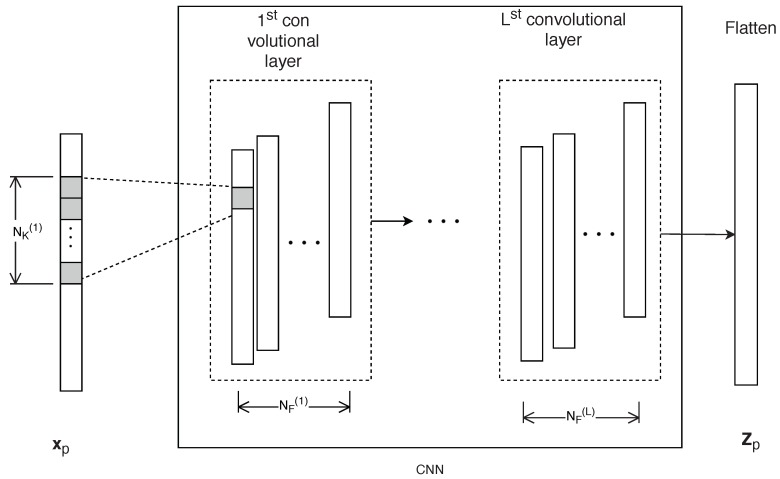
Proposed one-dimensional CNN model.

Figure 3 shows the proposed CNN model, that comprises one input vector, *L* convolutional layers, and one output vector with size $N_{C}\times N_{F}$ by a Flatten layer. The input vector for the pth packet transmission can be expressed as

(3) $$x_{p}=\left[R\left({\hat{H}}_{p}\left[-K/2\right]\right),I({\hat{H}}_{p}\left[-K/2\right],\ldots ,R\left({\hat{H}}_{p}\left[K/2\right]\right),I({\hat{H}}_{p}\left[-K/2\right]\right]$$

where ${\hat{H}}_{p}\left[k\right]$ is the CSI for the pth packet transmission, and R(.) and I(.) are the real and imaginary parts of the complex value, respectively. The lth convolutional layer convolves the input regions locally using $N_{F}$ filter kernels, where each filter uses the same kernel to extract the local features of the input region. The output of a convolution operation at the lth layer for one filter is determined by

(4) $$\begin{array}{c} y_{i}^{\left(l\right)}=a\left(\sum_{r=1}^{N_{K}^{\left(l\right)}}w_{r}^{\left(l\right)}x_{r+i\times N_{S}^{\left(l\right)}}+b^{\left(l\right)}\right),\\ 0\leq i\leq \frac{N-N_{K}^{\left(l\right)}}{N_{S}^{\left(l\right)}};l=1,2,\ldots ,L. \end{array}$$

where $N_{K}^{\left(l\right)}$ is the kernel size of the filters, $w_{r}^{\left(l\right)}$ and $b^{\left(l\right)}$ are the weight and bias elements located at (r) on the kernel, respectively, in the lth convolutional layer. In addition, a(.) represents a non-linearity activation function, that is typically given by the sigmoid, softsign, hyperbolic tangent (tanh) and rectified linear unit (reLU), etc. [31]. Without zero-padding, the output size is calculated as

(5) $$N_{C}^{\left(l\right)}=\frac{N_{C}^{\left(l-1\right)}-N_{K}^{\left(l\right)}}{N_{S}^{\left(l\right)}}+1,$$

where $N_{S}$ is a stride, which corresponds to how much a filter is shifted at a time. We put batch normalization (BN) after the non-linearity activation function applied after each CNN layer. The BN plays a role in regularization; its benefits are discussed in [32].

As shown in Figure 3, the CNN segment has *L* layers and we finally stress out the data to a vector with size $N_{C}\times N_{F}$ by a Flatten layer. Note that CSI data is different from actual images so when applying the CNN, we need to change some structures from the normal CNN model. The first thing is to set the stride step $N_{S}^{\left(1\right)}$ by even numbers (2,4, etc.) in the first convolutional layer to guarantee the characteristic of complex input data. Because the size of the CSI packet is small, the second difference is that we do not apply any pooling layers, thereby significantly reducing the size of the input, leading to the loss of some important information to training in the RNN segment.

### 4.2. RNN Part

**Figure 4:**
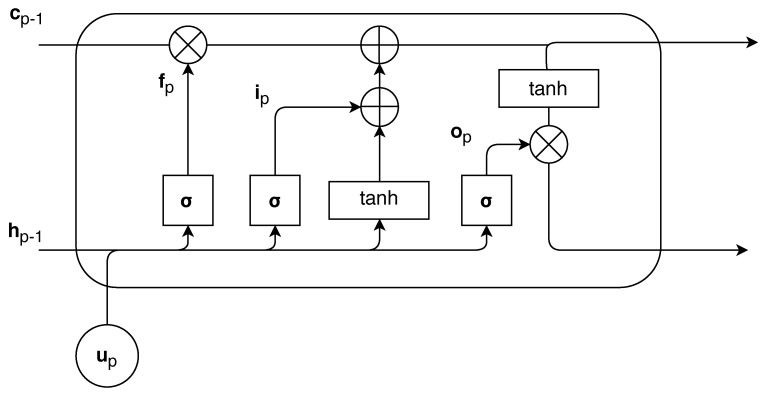
The structure of the LSTM.

The RNN model is composed of LSTM modules and an output layer for classification. The input vector for the LSTM module is defined as $u_{p}=\left[r_{p},z_{p}\right]$ where $r_{p}$ is an RSSI value for the pth packet transmission. The structure of the LSTM is shown in Figure 4. At the current time step *p*, the equations below describe the internal structure of the LSTM module:

(6) $$\left(\begin{array}{c} i_{p}\\ f_{p}\\ o_{p}\\ g_{p} \end{array}\right)=\left(\begin{array}{c} \sigma \\ \sigma \\ \sigma \\ tanh \end{array}\right)\left(\begin{array}{c} W_{ui}u_{p}+W_{ci}h_{p-1}+b_{i}\\ W_{uf}u_{p}+W_{cf}h_{p-1}+b_{f}\\ W_{uo}u_{p}+W_{co}h_{p-1}+b_{o}\\ W_{ug}u_{p}+W_{cg}h_{p-1}+b_{g} \end{array}\right)$$

(7) $$c_{p}=f_{p}⊙c_{p-1}+i_{p}⊙g_{p}$$

(8) $$h_{p}=o_{p}⊙tanh\left(c_{p}\right)$$

where $u_{p}$ is the input to the LSTM block; $i_{p}$, $f_{p}$, $o_{p}$, $c_{p}$ and $h_{p}$ are the input gate, the forget gate, the output gate, the cell state, and the output of the LSTM block, respectively. $W_{ui}$, $W_{uf}$, $W_{ug}$, and $W_{uo}$ are the weights between the cell state and the input gate, the forget gate, the external output gate, and the output gate, respectively. $W_{ci}$, $W_{cf}$, $W_{cg}$,and $W_{co}$ are the weights between the cell state and the input gate, the forget gate, the external output gate, and the output gate, respectively, and finally, $b_{i}$, $b_{f}$, $b_{g}$, and $b_{o}$ are the additive biases of the input gate, the forget gate, the external output gate, and the output gate, respectively. The sigmoid function σ(.) and the hyperbolic activation function tanh(.) are used as activation functions. In (7) and (8), the cell state, $c_{p}$, and the output of the LSTM block, $h_{p}$, are calculated using the outputs form the above gates in (6), where ⊙ denotes an element-wise multiplication.

Finally, for the NLOS and LOS condition decision, we put the feature vector $h_{P}$ extracted at the last LSTM cell through a single perceptron layer where *P* is the number of packet transmissions. The output $h_{\theta }$ of the model is calculated as follows:

(9) $$h_{\theta }=\sigma \left(Vh_{P}+b\right)$$

where V is the weight matrix that transfers the values in the Fully Connected (FC) layer to the output layer and *b* is a bias factor. In(9), the sigmoid function σ(.) is used to transform the logit of the single neuron in the final stage to calculate the probability for classifying the LOS or NLOS.

We set y=1 for LOS conditions and y=0 for NLOS conditions. During the training stage, at each epoch, we select multiple batches from the set of input and output pairs ([X,r],y) to train and verify the proposed CNNLSTM model. Every parameter in the model is adjusted to minimize the following lost function

(10) $$L=-\frac{1}{N}\sum_{g=1}^{N}C\left(g\right)$$

where *N* is the batch size of model, C(g) is the cost of the gth input and output pair that measures how accurately the model predicts the label that corresponds to the input. Among many choices of the loss function used in optimizing our model, we adopt the binary cross-entropy function, expressed by

(11) $$C\left(g\right)=y^{\left(g\right)}logh_{\theta }\left({\left[X,r\right]}^{\left(g\right)}\right)+\left(1-y^{\left(g\right)}\right)log(1-h_{\theta }\left({\left[X,r\right]}^{\left(g\right)}\right)$$

where the superscript is used to indicate the index of the input and output pair.

To minimize the loss function, many variants of the gradient-descent method such as AdaGrad, AdaDelta, and Adam optimizers have been studied. These optimizers adaptively change the learning rate to properly minimize the loss function. In this study, we applied the Adam optimization algorithm to train our proposed CNNLSTM model as the Adam optimizer is straight-forward and saves memory and computational resources.

The process starts with random initialization of all the model parameters. During the training phase, the weight update takes place after a whole sequence has been propagated forward through the network. The error signals are calculated with respect to the Mean of Cross Entropy Losses cost function. The loss function was chosen as the natural cost function for the sigmoid output layer with the aim of maximizing the likelihood of classifying the input data correctly.

Once every parameter in the proposed CNNLSTM model is adjusted appropriately, the model can identify the channel condition based on the following simple hypothesis test

(12) $$H_{0}:H_{\theta }\left(\left[X,r\right]\right)\geq \alpha$$

$$H_{1}:H_{\theta }\left(\left[X,r\right]\right)<\alpha$$

where $H_{0}$ and $H_{1}$ are null and alternative hypothesis, respectively, and α denotes the decision threshold. We assume that, LOS detection rate is a true positive rate (TPR) corresponding to the portion of correct decisions among all measurements under the LOS condition. Similarly, NLOS detection rate is a true negative rate (TNR) corresponding to the portion of correct decisions over all measurements. These statistical values depend on the decisions.

## 5. Performance Evaluation

In this section, we will discuss the results of the proposed scheme using CSI and RSSI data with a total 100,000 packet transmissions. We assess several numbers of packet transmissions, P=10, P=20, P=50 and P=100. We split our dataset into three parts: training, validation, and test sets. We use 70% of sample points to build our classification model during the training phase. 15% of data were used to compare the performances of the models in the validation phase. We selected the best model for the test phase. Finally, we applied our chosen model to the test set, the remaining 15% of the original data set, so as to evaluate how our model performs on unseen data. Note that, the test set was not used in the experiments.

The model was trained by truncated backpropagation through time [33] with Adam optimization [34] with an initial learning rate of 0.001. On the dataset, we used a minibatch [35] with a size of 128 for high efficiency. After each batch, the gradients were averaged and updated. We employed the early stopping method to stop the training when the validation accuracy becomes stagnant and does not increase after 10 epochs. We adopted the dropout method with a probability of 0.5 after the CNN layers and LSTM layer for regularization to avoid over-fitting problems [36].

In the CNN segment, we used hybrid hyper-parameters settings for the CNNs with high numbers, *L* of convolution layers to extract the implicit features of data. We applied the CNN models with a different number of $N_{F}^{\left(l\right)}$ filters, such as 16, 32, 64, 128, etc. with different kernel sizes, such as 2, 3, 4, etc., and with several kinds of popular activation functions, such as ReLU, tanh, sigmoid, etc. After testing out all the simulation settings, the best achieved model is as shown in Table 1. Here, we set number of CNN layers L=3 with kernel size, $N_{K}^{\left(1\right)}=8$ for the first layer, and $N_{K}^{\left(2\right)}=N_{K}^{\left(3\right)}=2$ for the second and third layer. In the first and second CNN layers, we use $N_{F}^{\left(1\right)}=32$ and $N_{F}^{\left(2\right)}=16$ filters with the softplus activation function. $N_{K}^{\left(3\right)}=2$ and $N_{F}^{\left(3\right)}=8$ are used for the third CNN layer.

In the RNN segment, we also set different the number of units in LSTM layer, $D_{h}$, such as 5, 10, 20, etc., to obtain the best result for our model.

**Figure 5:**
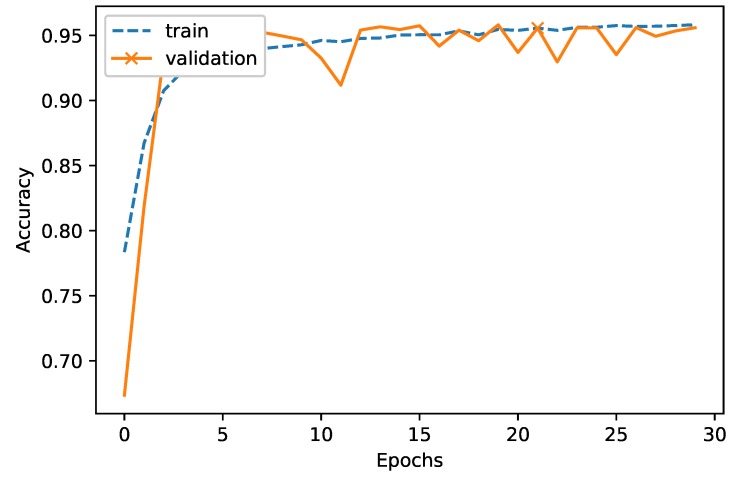
Performance convergence versus epochs.

Figure 5 shows the convergence of the model over epochs for the training and validation set. It can be seen that, the accuracy of the training set shows a trend of improvement in performance after each epoch. Conversely, the accuracy of the validation set decrease and fluctuates after reaching the top point of 96.32%. To avoid wasting time in training the data, we used the early stopping method that automatically stops the model if the accuracy of the validation set does not improve after several epochs (in our model, this value was set to 10). The peak point of the highest accuracy for the validation set occurred in epoch 21 and was marked by *X* symbol in the figure.

**Figure 6:**
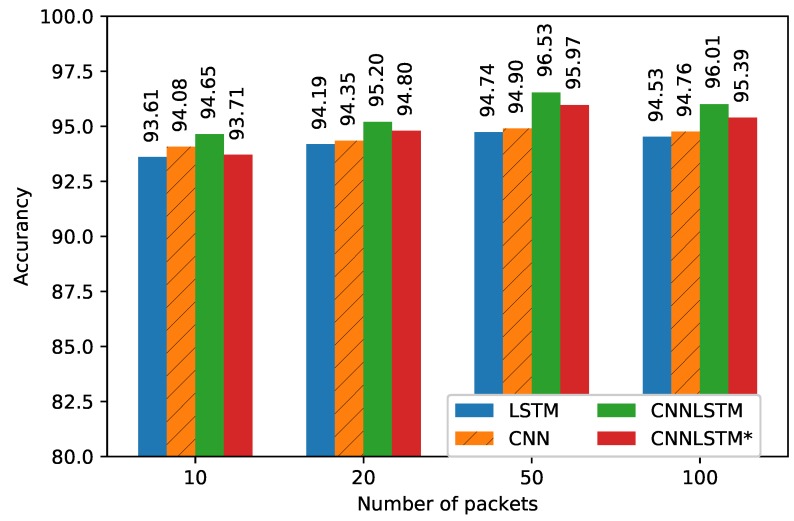
Performances of the models depend on the number of packets.

In this paper, we also implemented CNN methods and compared them with the conventional method LSTM method. The CNN model was also optimized similarly to the proposed CNN segment, except here, instead of the LSTM layer, we added the Flatten layer after the final CNN layer. The LSTM model only learns time relative sequence information whereas the CNN model focuses on extracting implicit features that contain space information, while our proposed model offers both of these advantages. Figure 6 illustrates the performance of the proposed model with various values of *P* for the test set. It can be seen that the best outcome was obtained at P=50. Even if *P* is increased to 100, the performance shows a decreasing trend for all models. Hence, in the results shown below, we use P=50 to compare to the building data.

Table 2 summarizes the performances decision thresholds that are selected to maximize the average LOS/NLOS detection rate for the LSTM, CNN, and CNNLSTM models. As can be seen, the CNNLSTM model outperforms the other models in both accuracy and average detection rate for all values of *P*. Note that CNNLSTM* denotes a model that applies the absolute values of CSI data. It offers slightly better performance than the LSTM and CNN models while using simpler data, so we can consider it as a choice when generating data from practical instruments. The input signal for the CNN segment in this case can be written as

(13) $$x_{p}^{⋆}={\left[|{\hat{H}}_{p}\left[-K/2\right]|,\ldots ,|{\hat{H}}_{p}\left[K/2\right]|\right]}^{T}$$

where $|{\hat{H}}_{p}\left[k\right]|$ is the amplitude value of the CSI for the $p^{th}$ packet transmission.

Table 3 shows the total training time and the number of parameters of the LSTM, CNN and proposed CNNLSTM models, where an NVIDIA Titan X GPU 1.4 GHz with 3584 cores is used for simulations. The total training time for the proposed CNNLSTM is comparable to the CNN and LSTM models because the proposed model takes only 21 epochs to converge to the optimal solution. The number of parameters for the proposed CNNLSTM is larger than the LSTM model. However, the memory requirement is less demanding in the test time evaluation because there is no backward propagation. In addition, the proposed CNNLSTM* reduces the total training time and number of parameters compared to the proposed CNNLSTM.

**Figure 7:**
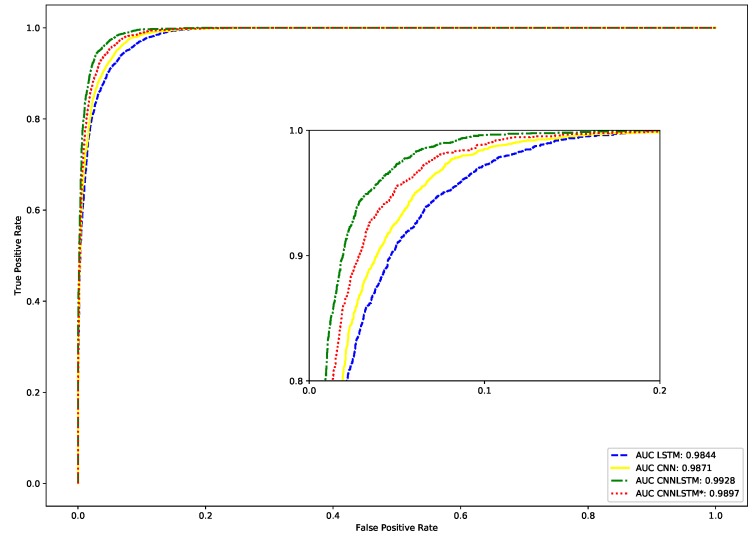
ROC curves of LOS identification using the proposed CNNLSTM and other methods.

In Figure 7, we use the receiver operating characteristic (ROC) curve, that describes the relationship between TPR and FPN. If the performance is better, the ROC curve will approach a point in the upper left edge, that implies perfect discrimination. As we can see, the LSTM model has worse performance. Conversely, the proposed CNNLSTM model offers the best result because its area under curve (AUC) of 0.9928 approximates with perfect result of 1 [18,19,37].

**Figure 8:**
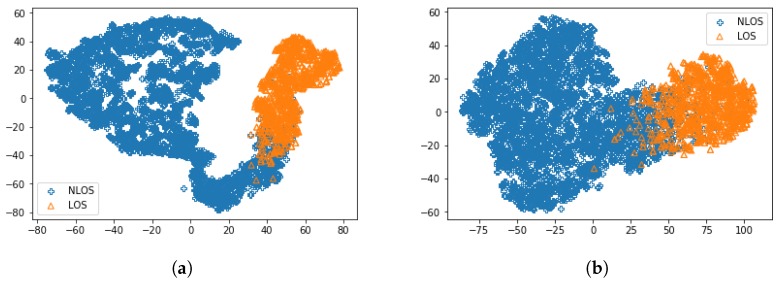
tSNE representation of 5000 training samples for (**a**) proposed CNNLSTM and (**b**) conventional LSTM.

To further understand what is inside the model, we analyze the internal representations of the trained network. Following the training procedure, the hidden state vector of the last LSTM module was used to visualize the trained network. Figure 8 shows t-SNE representations using 5000 different inputs from the training set of the proposed method and conventional LSTM method [16], where t-SNE aims to project the high dimensional vectors to two-dimensional space while retaining their pairwise similarity [38]. In the figure, we can see that the hidden state for the proposed method was much more dispersed compared to the hidden state for the conventional method [16]. This explains the improved accuracy based on feature extraction by using CNN segment in the proposed CNNLSTM.

## 6. Conclusions

In this paper, we proposed a deep learning model to identify channel conditions by combining CNN and RNN. In the proposed CNNLSTM model, the CNN captured the feature from frequency-domain characteristics of CSIs and then LSTMs extracted the temporal feature from RSSI and the output of CNN. In addition, a CNNLSTM model with the absolute value of CSIs was proposed to reduce the complexity with slightly better performance than the conventional models. The proposed methods were verified under indoor environments for WLANs and achieved higher accuracy than the conventional LSTM model in classifying LOS and NLOS. In future work, we would like to investigate the performance of the proposed CNNLSTM models in outdoor environments to expand the range of applications of the algorithm.

## Figures and Tables

**Table 1:** Details of proposed CNNLSTM model.

Layer Type	Activation	Kernel Size	Stride	Filter	Output Shape
Input					50 × 112 × 1
Convolution	softplus	8	2	32	50 × 53 × 32
Convolution	softplus	2	1	16	50 × 52 × 16
Convolution	reLU	2	1	8	50 × 51 × 8
Flatten	_	_	_	_	50 × 408
Concatenate with RSSI	_	_	_	_	50 × 409
LSTM	_	_	_	10	10
FC	sigmoid	_	_	1	1

**Table 2:** Performances according to the models.

Model	Decision Threshold	Avg Detection Rate	Accuracy
LSTM	0.332970	0.94920	94.57
CNN	0.372340	0.94310	94.90
CNNLSTM	0.302848	**0.95989**	**96.53**
CNNLSTM*	0.225334	0.95141	95.97

**Table 3:** The training time and number of parameters of the models.

Model	Time (s)	Epochs	Total Training Time (s)	Number of Parameters
LSTM	**9**	37	333	5011
CNN	16	25	400	22,463
CNNLSTM	17.5	**21**	367.5	18,863
CNNLSTM*	15	23	345	9679
